# Vasohibin-2 promotes proliferation in human breast cancer cells via upregulation of fibroblast growth factor-2 and growth/differentiation factor-15 expression

**DOI:** 10.3892/mmr.2014.2317

**Published:** 2014-06-10

**Authors:** MIN TU, XIAN LIU, BEI HAN, QIANQIAN GE, ZHANJUN LI, ZIPENG LU, JISHU WEI, GUOXIN SONG, BAOBAO CAI, NAN LV, KUIRONG JIANG, SHUI WANG, YI MIAO, WENTAO GAO

**Affiliations:** 1 Department of General Surgery, The First Affiliated Hospital of Nanjing Medical University, Nanjing, Jiangsu 210029, P.R. China; 2 Department of Endocrinology, Nanjing Children’s Hospital Affiliated to Nanjing Medical University, Nanjing, Jiangsu 210008, P.R. China; 3 Department of Pathology, The First Affiliated Hospital of Nanjing Medical University, Nanjing, Jiangsu 210029, P.R. China

**Keywords:** vasohibin-2, breast cancer, proliferation, growth factor

## Abstract

Vasohibin-2 (VASH2) is an angiogenic factor, and has been previously reported to be a cancer-related gene, with cytoplasmic and karyotypic forms. In the current study VASH2 expression in human breast cancer tissue and adjacent non-cancerous tissue was investigated with immunohistochemistry. MCF-7 and BT474 human breast cancer cells were transfected with lentiviral constructs to generate *in vitro* VASH2 overexpression and knockdown models. In addition, BALB/cA nude mice were inoculated subcutaneously with transfected cells to generate *in vivo* models of VASH2 overexpression and knockdown. The effect of VASH2 on cell proliferation was investigated using a bromodeoxyuridine assay *in vitro* and immunohistochemistry of Ki67 in xenograft tumors. Growth factors were investigated using a human growth factor array, and certain factors were further confirmed by an immunoblot. The results indicated that the expression level of cytoplasmic VASH2 was higher in breast cancer tissues with a Ki67 (a proliferation marker) level of ≥14%, compared with tissues with a Ki67 level of <14%. VASH2 induced proliferation *in vitro* and *in vivo*. Four growth factors activated by VASH2 were identified as follows: Fibroblast growth factor 2 (FGF2), growth/differentiation factor-15 (GDF15), insulin-like growth factor-binding protein (IGFBP)3 and IGFBP6. FGF2 and GDF15 may contribute to VASH2-induced proliferation. The current study identified a novel role for VASH2 in human breast cancer, and this knowledge suggests that VASH2 may be a novel target in breast cancer treatment.

## Introduction

The vasohibin (VASH) family consists of two members, VASH1 and VASH2 (1). VASH1 was initially identified as a regulator of negative feedback in angiogenesis induced by vascular endothelial growth factor (VEGF) or fibroblast growth factor 2 (FGF2) (2,3). VASH2 is a VASH1 homolog expressed in mononuclear cells that has been demonstrated to act as an angiogenesis stimulator in a mouse model of hypoxia-induced subcutaneous angiogenesis (3). VASH2 is also involved in the proliferation of hepatic (4) and ovarian (5,6) cancer.

It was previously demonstrated that there are two types of VASH2: Nuclear and cytoplasmic (7). In the present study, the focus was on cytoplasmic VASH2, thus all subsequent mention of VASH2 refers to the cytoplasmic form. VASH2 expression was investigated in human breast cancer in the current study; rabbit polyclonal anti-human VASH2 antibodies were produced and successfully used in immunoblotting and immunohistochemical analysis (7).

In the present study, VASH2 expression levels were indicated to be higher in grade 3 vs. grade 1–2 tissues, and in tissues with a level of Ki67 ≥14%. Ki67 is a marker for breast cancer proliferation. It was hypothesized that VASH2 is associated with cell proliferation in breast cancer, and in order to investigate the proliferative function of VASH2 in breast cancer cells and the underlying mechanism, VASH2 overexpression and knockdown *in vitro* and *in vivo* models were established. VASH2 produced a significant proliferative effect *in vitro* and *in vivo*. Human growth factor array demonstrated that VASH2 promoted proliferation in breast cancer cells via the upregulation of FGF2 and growth/differentiation factor-15 (GDF15) expression. The present study identified a novel role for VASH2 in human breast cancer, and this knowledge may lead to the possibility of VASH2 as a novel target in breast cancer treatment.

## Materials and methods

### Clinical samples

Human breast cancer tissue and adjacent non-cancerous tissue were obtained from 99 patients who underwent surgical resection at The First Affiliated Hospital of Nanjing Medical University (Nanjing, China) in accordance with institutional policy. All patients provided written informed consent.

### Animals

Five-week-old female BALB/cA-nu (nu/nu) nude mice were obtained from Vital River Laboratories (Beijing, China). The Animal Care and Use Subcommittee of Nanjing Medical University approved all experimental procedures, which were performed in accordance with the standards established by the 1964 Declaration of Helsinki and its later amendments. Animals were sacrificed using pure carbon dioxide.

### Cell culture

The MCF-7 human breast cancer cell line was obtained from the Shanghai Cell Bank (Type Culture Collection Committee, Chinese Academy of Sciences, Shanghai, China) and cultured according to the manufacturer’s instructions. The BT474 human breast cancer cell line was provided by Professor Shui Wang of the Department of General Surgery, The First Affiliated Hospital of Nanjing Medical University (Nanjing, China) and cultured in complete Dulbecco’s modified Eagle’s medium (Gibco, Carlsbad, CA, USA) supplemented with 10% fetal bovine serum (Gibco). Cells were cultivated in a humidified 5% CO_2_ incubator at 37°C.

### Plasmid construction and lentivirus packaging

Lentiviral (Lv) constructs were designed to induce VASH2 overexpression and knockdown as previously described (4). MCF-7 cells were stably transfected with Lv-CMV-VASH2 for VASH2 overexpression and termed MCF7-VASH2; MCF-7 cells stably transfected with Lv-CMV-enhanced green fluorescent protein (EGFP) for VASH2 knockdown were termed MCF7-EGFP; BT474 cells stably transfected with VASH2-targeting short hairpin RNA (shRNA) lentivirus for VASH2 knockdown were termed BT474-shVASH2; and BT474 cells stably transfected with scrambled shRNA lentivirus as controls were termed BT474-scramble.

### Immunoblotting

Whole cell lysates were prepared in radioimmunoprecipitation assay buffer (Beyotime, Nantong, China) and blotted using the following primary antibodies: Rabbit polyclonal anti-VASH2 [prepared as described in (7)]; rabbit polyclonal anti-FGF2 (Sigma-Aldrich, St. Louis, MO, USA); goat polyclonal anti-GDF15 (Santa Cruz Biotechnology, Santa Cruz, CA, USA); and mouse monoclonal anti-GAPDH (Beyotime, Nantong, China). The secondary antibodies used for detection were horseradish peroxidase (HRP)-conjugated goat anti-mouse immunoglobulin G (IgG) (CWbio, Shanghai, China) and HRP-conjugated donkey anti-rabbit IgG (CWbio).

### Immunohistochemistry

Immunohistochemical staining of the clinical samples was performed as previously described (7). Xenograft tumors were harvested from mice and stained with the following primary antibodies: Rabbit polyclonal anti-VASH2 [prepared as previously described (7)], rabbit polyclonal anti-estrogen receptor (ER)α and Ki67 (Maixin Biotech, Fuzhou, China), and mouse monoclonal antibodies targeting the progesterone receptor (PR) and human epidermal growth factor receptor 2 (HER2; Maixin Biotech). VASH2 staining intensity was classified as weak or strong. ERα, PR and HER2 staining were classified as positive or negative. HER2^+/++^ was also classified as negative; only HER2^+++^ was classified as positive. Ki67 staining was classified as <14% and ≥14%. VASH2 staining intensity was classified as Low (negative or weak staining) and high (middle or strong staining).

### In vivo tumorigenesis

MCF7-EGFP or MCF7-VASH2 cells (2×10^6^) were bilaterally injected subcutaneously into the flanks of eight mice. Eighty days later, the mice were sacrificed and the xenograft tumors harvested. In addition, BT474-scramble or BT474-shVASH2 cells (1×10^6^) were bilaterally injected subcutaneously into the flanks of seven mice and the xenograft tumors were harvested at 60 days post-inoculation. Tumor volume was calculated as follows: (Width^2^ × length)/2.

### Bromodeoxyuridine (BrdU) proliferation assay

MCF7-EGFP, MCF7-VASH2 (2×10^3^ cells/well), BT474-scramble and BT474-shVASH2 cells (3×10^3^ cells/well) were seeded in 96-well tissue culture plates. Eight wells were used for each cell type. At 48 h following seeding, cell proliferation enzyme-linked immunosorbent assay (ELISA) using a BrdU kit (cat no. 11647229001; Roche, Mannheim, Germany) was performed according to the manufacturer’s instructions.

### Antibody array

Quantitative sandwich-based antibody array (#QAH-GF-1; RayBiotech, Guangzhou, China) was used to detect 40 human growth factors in lysates of the MCF7-EGFP, MCF7-VASH2, BT474-scramble and BT474-shVASH2 cells. All detection services were provided by RayBiotech (Norcross, GA, USA). Each antibody produced four dots, and the averages of the median signal intensities were used for all calculations. Fold change of >1.5 or <0.66 compared with controls was selected as the distinction between overexpression or knockdown, respectively.

### Statistical analysis

Statistical analysis was performed using SPSS, version 13.0 (SPSS, Inc., Chicago, IL, USA). Comparisons between treated and control groups were conducted using Student’s t-test, and P<0.05 was considered to indicate a statistically significant difference. Pearson’s χ^2^ test was used to compare rates of the data in Table I.

## Results

### VASH2 staining and the clinicopathological characteristics of breast cancer

**Figure 1:**
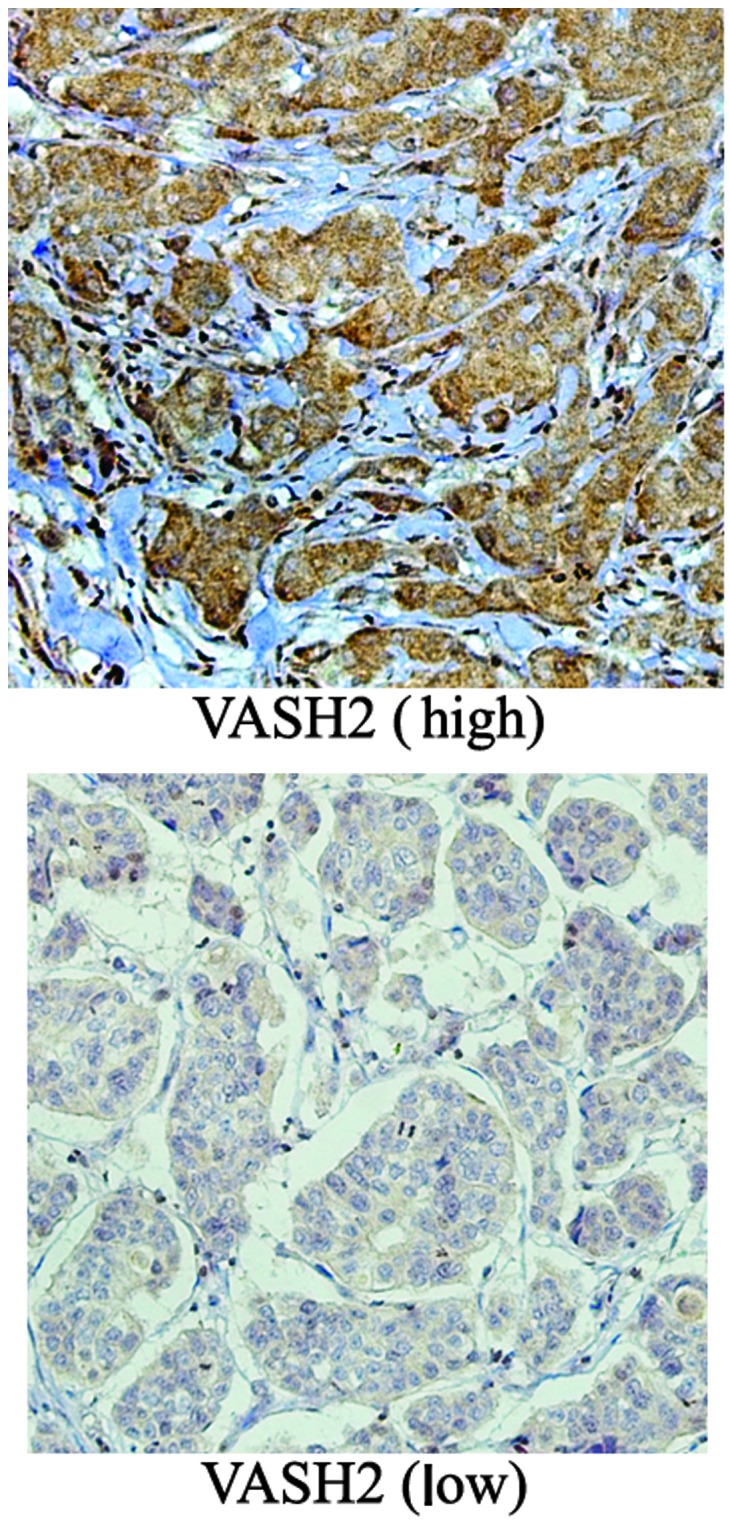
Representative images of VASH2 staining in human breast cancer tissues. Magnification, ×200. VASH2, vasohibin-2.

VASH2 expression levels in 99 human breast cancer tissue samples were assessed by immunohistochemical analysis. Table I shows the association between VASH2 staining and the clinicopathological characteristics of breast cancer. VASH2 expression was high in 70/99 (70.7%) breast cancer tissues. VASH2 staining was generally higher in grade 3 tissues and those with Ki67 ≥14% (Pearson χ^2^, P<0.001). These findings indicate that VASH2 may promote proliferation in human breast cancer cells. Fig. 1 displays the representative images of high and low VASH2 expression levels in human breast cancer tissues.

### VASH2 promotes proliferation in human breast cancer cells in vitro and in vivo

**Figure 2:**
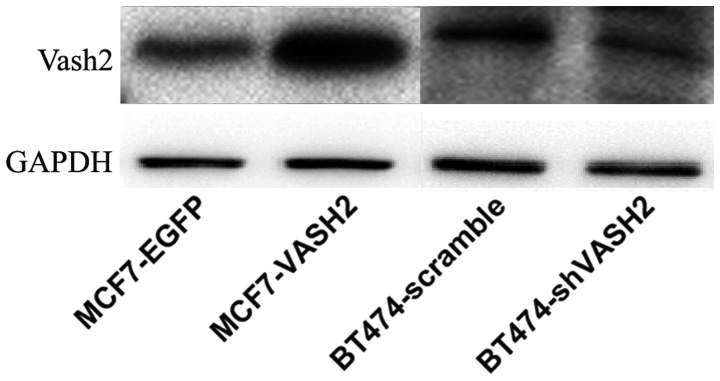
VASH2 expression in stably transfected MCF-7 and BT474 cells. MCF-7 cells were transfected with a vector expressing EGFP (MCF7-EGFP) or VASH2 (MCF7-VASH2); BT474 cells were transduced with scrambled shRNA (BT474-scramble) or VASH2-targeting shRNA (BT474-shVASH2). The level of VASH2 protein in each cell type was assessed by immunoblotting. GAPDH, glyceraldehyde-3-phosphate dehydrogenase; VASH2, vasohibin-2; EGFP, enhanced green fluorescent protein; shRNA, short hairpin RNA.

**Figure 3:**
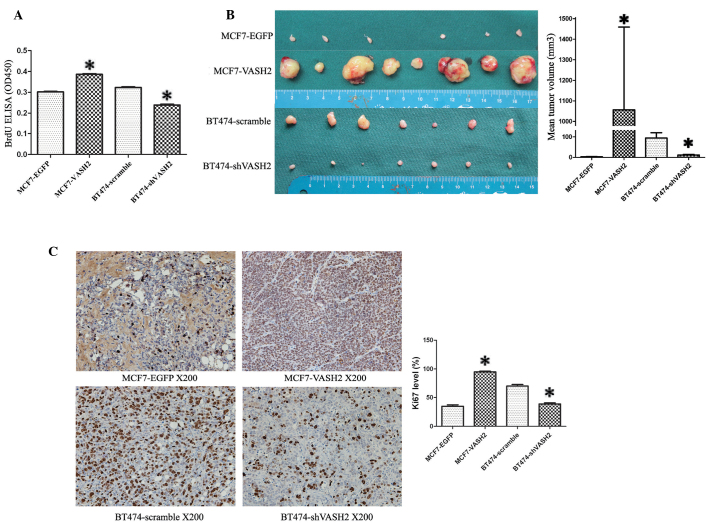
VASH2 induced proliferation in human breast cancer cells *in vitro* and *in vivo*. (A) *In vitro* effects of VASH2 on cell proliferation measured by BrdU incorporation, which was measured using ELISA. Absorbance was read at 450 nm (^*^P<0.05, n=8). (B) Xenograft tumors from mice injected subcutaneously with MCF7-EGFP, MCF7-VASH2, BT474-scramble or BT474-shVASH2 cells. The data are presented as the mean ± standard error of tumor volume of each group. MCF7-EGFP (2.8±1.1 mm^3^) vs. MCF7-VASH2 (1057.0±402.8 mm^3^), ^*^P<0.05, n=8; BT474-scramble (94.4±25.5 mm^3^) vs. BT474-shVASH2 (11.3±3.3 mm^3^), ^#^P<0.05, n=7. (C) Immunohistochemistry of Ki67 in xenograft tumors. The data presented are the average Ki67 level ± standard error (%) of tumors for each group. MCF7-EGFP (34.8±2.5) vs. MCF7-VASH2 (95.0±1.2), ^*^P<0.05; BT474-scramble (69.8±2.8) vs. BT474-shVASH2 (33.8±1.8), ^#^P<0.05. BrdU, bromodeoxyuridine; OD, optical density; EGFP, enhanced green fluorescent protein; VASH2, vasohibin-2.

The VASH2 expression levels in VASH2-overexpressing (MCF7-VASH2) and VASH2 knockdown cells (BT474-shVASH2) were confirmed using immunoblotting (Fig. 2), indicating the successful establishment of *in vitro* models of VASH2 overexpression and knockdown. The proliferative function of VASH2 was investigated *in vitro* using cell proliferation ELISAs. Results indicated that the optical density at 450 nm (OD450) of MCF7-VASH2 cells was significantly higher than that of MCF7-EGFP cells, while the OD450 of BT474-shVASH2 cells was significantly lower than that of BT474-scramble cells (Fig. 3A, P<0.05). These data indicate that VASH2 induced cell proliferation *in vitro*.

MCF7-EGFP, MCF7-VASH2, BT474-scramble or BT474-shVASH2 cells were injected into the flanks of nude mice. At 80 days post-inoculation, mice that had been injected with MCF7-VASH2 cells had developed significantly larger tumors than mice injected with MCF7-EGFP cells (Fig. 3B, P<0.05). At 60 days post-inoculation, mice that had been injected with BT474-shVASH2 cells had developed significantly smaller tumors than mice injected with BT474-scramble cells (Fig. 3B, P<0.05). The levels of Ki67 staining in MCF7-VASH2 xenograft tumors were significantly higher than in MCF7-EGFP xenograft tumors (Fig. 3C, P<0.05), and the levels in BT474-shVASH2 xenograft tumors were significantly lower than in BT474-scramble xenograft tumors (Fig. 3C, P<0.05). These findings indicate that VASH2 also induces proliferation *in vivo*.

### VASH2 induces FGF2, GDF15, insulin-like growth factor-binding protein (IGFBP)3 and IGFBP6 expression

**Figure 4:**
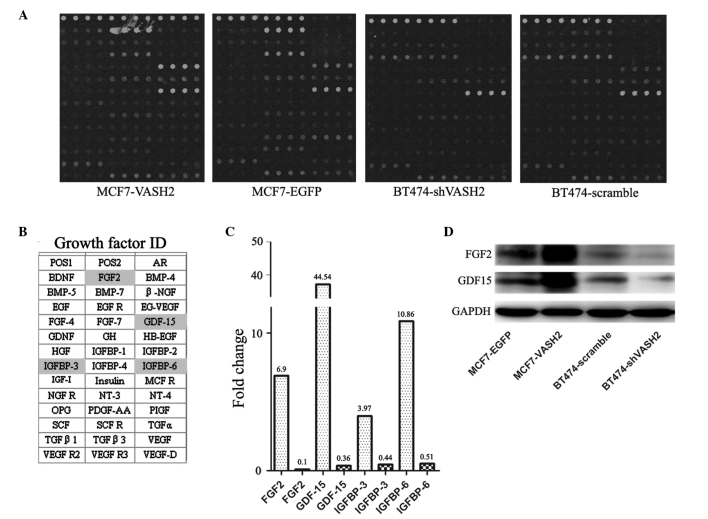
Human growth factor array and verification of positive hits. (A) Dots on human growth factor antibody arrays. (B) Names of the tested angiogenic factors. Growth factor fold changes of >1.5 in VASH2-overexpressing cells or <0.66 in VASH2 knockdown cells were selected as up- and downregulated, respectively (highlighted). (C) Distinct fold change of four growth factors: FGF2, GDF15, IGFBP3 and IGFBP6. (D) Immunoblot analysis of FGF2 and GDF15. VASH2; vasohibin-2; EGFP, enhanced green fluorescent protein; AR, androgen receptor; BDNF, brain-derived neurotrophic factor; FGF, fibroblast growth factor; BMP, bone morphogenetic protein; EGF, epidermal growth factor; GDF, growth/differentiation factor; HB-EGF, heparin binding-EGF; IGFBP, insulin-like growth factor-binding protein; VEGF, vascular endothelial growth factor.

Using sandwich-based antibody array, 40 human growth factors were detected in MCF7-EGFP, MCF7-VASH2, BT474-scramble and BT474-shVASH2 cell lysate samples. Table II denotes the growth factor array results. A fold change of >1.5 or <0.66 compared with controls was selected as the definition of up- and downregulation, respectively. FGF2, GDF15, IGFBP3 and IGFBP6 expression levels were elevated in the VASH2-overexpressing MCF7-VASH2 cells and were reduced in the VASH2-knockdown BT474-shVASH2 cells; this indicates that they may have contributed to the specific reaction induced by VASH2 (Fig. 4). The protein expression levels of FGF2 and GDF15 were then detected by immunoblot, which confirmed the results of the antibody array (Fig. 4D). Androgen receptor, brain-derived neurotrophic factor, bone morphogenetic protein-4, epidermal growth factor (EGF), heparin-binding EGF, IGFBP2, and VEGF receptor-2 fold changes were also >1.5 (Table II) in the VASH2-overexpressing MCF7-VASH2 cells, but were not altered in VASH2-knockdown BT474-shVASH2 cells. Notably, the fold changes for platelet-derived growth factor-AA and placental growth factor in the VASH2-overexpressing and knockdown cells were <0.66 (Table II), indicating that they may have contributed to a non-specific reaction. These data suggest that VASH2 upregulates FGF2, GDF15, IGFBP3 and IGFBP6 expression.

## Discussion

VASH2 is involved in tumor proliferation (4,5). Rabbit anti-human VASH2 polyclonal antibodies were generated and used in immunohistochemical analysis of VASH2 expression levels (7). In the present study, VASH2 expression in clinical human breast cancer tissues was investigated, and significantly higher levels of VASH2 in grade 3 and Ki67 ≥14% breast cancer tissues were detected. Ki67 expression levels vary during the cell cycle; the levels are low during the G_1_ and early S phases, and high during mitosis, followed by a sharp reduction during anaphase and telophase. Ki67 cannot be detected during the G_0_ resting phase (8,9). Ki67 inhibition leads to the arrest of cell proliferation (10,11). Ki67 expression levels increase progressively from benign breast disease to ductal carcinoma *in situ* to invasive breast cancer (12–14). In addition, Ki67 is considered to be a good proliferation marker in clinical practice (15). In the current study, it was hypothesized that VASH2 is associated with cell proliferation, and to confirm the possible function of VASH2 in proliferation, *in vitro* and *in vivo* models of VASH2 overexpression and knockdown were developed. Analysis of the models indicated that VASH2 promotes the proliferation of breast cancer cells *in vitro* and *in vivo*.

Various cancer cells synthesize growth factors to which they are responsive (16), and these growth factors are important in the processes of tumor cell clonal expansion, angiogenesis, invasion and metastasis (17). It was hypothesized that VASH2 may induce proliferation via activation of growth factor expression. To confirm this, human growth factor array analysis was performed using VASH2-overexpression and knockdown *in vitro* models. A total of 40 common proliferation-related growth factors in four cell lysate samples (MCF7-VASH2, MCF7-EGFP, BT474-shVASH2 and BT474-scramble) were investigated. VASH2 increased the expression of four growth factors: FGF2, GDF15, IGFBP3 and IGFBP6. FGF2 (18) induces cell proliferation in various types of cancer. GDF15 serves a function in cell proliferation, apoptosis, metastasis and angiogenesis, through autocrine and paracrine signaling (19). IGFBP3 and IGFBP6 are IGF-binding proteins that inhibit IGFs, therefore functioning as tumor suppressors (20,21). However, IGFBP3 overexpression in breast cancer is linked to poor prognosis (22,23). Previously, it has been reported that IGFBP3 promotes cancer cell growth via an IGF-independent manner (24). It was also reported that IGFBP6 promoted cancer cell migration in an IGF-independent manner (21). Therefore, the function of VASH2-regulated IGFBP3 and IGFBP6 expression remains unclear. It is possible that the VASH2-induced proliferation occurred via upregulation of the expression of FGF2 and GDF15.

The present study demonstrated a high level of VASH2 expression in breast cancer cells, and that VASH2 functions as an inducer of growth factor expression, promoting cell proliferation in breast cancer. In conclusion, the current study indicated that VASH2 may have potential as a novel anticancer target.

## Figures and Tables

**Table I:** Association of VASH2 staining with clinicopathological characteristics of breast cancer.

Clinicopathologic characteristic	No. of patients	VASH2 staining		P-value

		Low (%)	High (%)	
Age				0.344
< 45	27	6 (22.3)	21 (77.7)	
≥45	72	23 (31.9)	49 (68.1)	
Pathological stage				0.299
T1	50	17 (34.0)	33 (66.0)	
T2–4	49	12 (24.5)	37 (75.5)	
Tumor grade				<0.001
G1–2	66	27 (40.9)	39 (59.1)	
G3	33	2 (6.1)	31 (93.9)	
Node status				0.952
Negative	61	18 (29.5)	43 (70.5)	
Positive	38	11 (28.9)	27 (71.1)	
ERα status				0.066
Negative	34	6 (17.6)	28 (82.4)	
Positive	65	23 (35.4)	42 (64.6)	
PR status				0.734
Negative	47	13 (27.7)	34 (72.3)	
Positive	52	16 (30.8)	36 (69.2)	
HER-2 status				0.988
Negative	75	22 (29.3)	53 (70.7)	
Positive	24	7 (29.2)	17 (70.8)	
Ki67 status				<0.001
<14%	28	19 (67.9)	09 (32.1)	
≥14%	71	10 (14.1)	61 (85.9)	
Total	99	29 (29.3)	70 (70.7)	

VASH2, vasohibin-2; ER, estrogen receptor; HER, human epidermal growth factor receptor. VASH2 staining: Low, negative or weak staining; High, middle or strong staining.

**Table II:** Human growth factor array results (#QAH-GF-1; RayBiotech).

Growth factor ID	MCF7-EGFP	MCF7-VASH2	BT474-scramble	BT474-shVASH2	MCF7-VASH2/MCF7-EGFP (fold change)	BT474-shVASH2/BT474-scramble (fold change)
POS1	31828	31621	31798	31686	0.99	1
POS2	8172	8225	8180	8209	1.01	1
AR	124	3345	154	135	26.92	0.88
BDNF	71	268	121	94	3.8	0.78
FGF2	5539	38221	255	27	6.9	0.1
BMP-4	43	126	48	43	2.91	0.89
BMP-5	175	226	205	220	1.29	1.07
BMP-7	202	282	207	220	1.4	1.06
β-NGF	45	35	44	31	0.78	0.71
EGF	22	58	1037	909	2.67	0.88
EGF R	1237	922	1024	629	0.75	0.61
EG-VEGF	39	57	39	52	1.46	1.33
FGF-4	55	75	45	31	1.35	0.7
FGF-7	54	81	47	45	1.49	0.96
GDF-15	783	34885	418	151	44.54	0.36
GDNF	110	113	42	62	1.02	1.48
GH	318	351	413	283	1.1	0.68
HB-EGF	262	393	269	324	1.5	1.21
HGF	47	41	28	26	0.87	0.92
IGFBP-1	102	148	104	70	1.45	0.67
IGFBP-2	2357	5127	3863	4603	2.18	1.19
IGFBP-3	63	249	63	28	3.97	0.44
IGFBP-4	68	97	77	95	1.44	1.24
IGFBP-6	16	170	47	24	10.86	0.51
IGF-I	36	53	30	22	1.47	0.72
Insulin	232	289	259	258	1.25	1
MCF R	135	161	133	111	1.19	0.84
NGF R	121	137	126	137	1.14	1.09
NT-3	70	102	53	42	1.46	0.79
NT-4	70	101	48	46	1.45	0.95
OPG	38	30	36	29	0.78	0.8
PDGF-AA	299	195	134	56	0.65	0.42
PIGF	570	122	199	120	0.21	0.6
SCF	70	100	48	51	1.43	1.07
SCF R	356	195	172	161	0.55	0.94
TGFα	27	32	18	21	1.21	1.16
TGFβ1	376	532	414	435	1.41	1.05
TGFβ3	44	64	50	37	1.45	0.73
VEGF	156	160	117	92	1.02	0.79
VEGF R2	42	76	34	28	1.81	0.83
VEGF R3	656	809	774	789	1.23	1.02
VEGF-D	305	431	338	331	1.41	0.98

EGFP, enhanced green fluorescent protein; VASH2, vasohibin-2; AR, androgen receptor; BDNF, brain-derived neurotrophic factor; FGF, fibroblast growth factor; BMP, bone morphogenetic protein; EGF, epidermal growth factor; GDF, growth/differentiation factor; HB-EGF, heparin binding-EGF; IGFBP, insulin-like growth factor-binding protein; VEGF, vascular endothelial growth factor.

## References

[1] Sato Y (2013). The vasohibin family: a novel family for angiogenesis regulation. J Biochem.

[2] Shibuya T, Watanabe K, Yamashita H, Shimizu K, Miyashita H, Abe M, Moriya T, Ohta H, Sonoda H, Shimosegawa T, Tabayashi K, Sato Y (2006). Isolation and characterization of vasohibin-2 as a homologue of VEGF-inducible endothelium-derived angiogenesis inhibitor vasohibin. Arterioscler Thromb Vasc Biol.

[3] Kimura H, Miyashita H, Suzuki Y, Kobayashi M, Watanabe K, Sonoda H, Ohta H, Fujiwara T, Shimosegawa T, Sato Y (2009). Distinctive localization and opposed roles of vasohibin-1 and vasohibin-2 in the regulation of angiogenesis. Blood.

[4] Xue X, Gao W, Sun B, Xu Y (2013). Vasohibin 2 is transcriptionally activated and promotes angiogenesis in hepatocellular carcinoma. Oncogene.

[5] Takahashi Y, Koyanagi T, Suzuki Y, Saga Y, Kanomata N, Moriya T, Suzuki M, Sato Y (2012). Vasohibin-2 expressed in human serous ovarian adenocarcinoma accelerates tumor growth by promoting angiogenesis. Mol Cancer Res.

[6] Koyanagi T, Suzuki Y, Saga Y, Machida S, Takei Y, Fujiwara H, Suzuki M, Sato Y (2013). In vivo delivery of siRNA targeting vasohibin-2 decreases tumor angiogenesis and suppresses tumor growth in ovarian cancer. Cancer Sci.

[7] Sun J, Tu M, Han B, Xue X (2013). Generation and characterization of rabbit polyclonal antibodies against Vasohibin-2 for determination of its intracellular localization. Int J Oncol.

[8] Beresford MJ, Wilson GD, Makris A (2006). Measuring proliferation in breast cancer: practicalities and applications. Breast Cancer Res.

[9] Lopez F, Belloc F, Lacombe F, Dumain P, Reiffers J, Bernard P, Boisseau MR (1991). Modalities of synthesis of Ki67 antigen during the stimulation of lymphocytes. Cytometry.

[10] Verheijen R, Kuijpers HJ, Schlingemann RO, Boehmer AL, van Driel R, Brakenhoff GJ, Ramaekers FC (1989). Ki-67 detects a nuclear matrix-associated proliferation-related antigen. I. Intracellular localization during interphase. J Cell Sci.

[11] Heidebrecht HJ, Buck F, Haas K, Wacker HH, Parwaresch R (1996). Monoclonal antibodies Ki-S3 and Ki-S5 yield new data on the ‘Ki-67’ proteins. Cell Prolif.

[12] Allred DC, Mohsin SK, Fuqua SA (2001). Histological and biological evolution of human premalignant breast disease. Endocr Relat Cancer.

[13] Rudas M, Neumayer R, Gnant MF, Mittelböck M, Jakesz R, Reiner A (1997). p53 protein expression, cell proliferation and steroid hormone receptors in ductal and lobular in situ carcinomas of the breast. Eur J Cancer.

[14] Shoker BS, Jarvis C, Davies MP, Iqbal M, Sibson DR, Sloane JP (2001). Immunodetectable cyclin D(1)is associated with oestrogen receptor but not Ki67 in normal, cancerous and precancerous breast lesions. Br J Cancer.

[15] Kontzoglou K, Palla V, Karaolanis G, Karaiskos I, Alexiou I, Pateras I, Konstantoudakis K, Stamatakos M (2013). Correlation between Ki67 and breast cancer prognosis. Oncology.

[16] Sporn MB, Todaro GJ (1980). Autocrine secretion and malignant transformation of cells. N Engl J Med.

[17] Witsch E, Sela M, Yarden Y (2010). Roles for growth factors in cancer progression. Physiology (Bethesda).

[18] Chandler LA, Sosnowski BA, Greenlees L, Aukerman SL, Baird A, Pierce GF (1999). Prevalent expression of fibroblast growth factor (FGF) receptors and FGF2 in human tumor cell lines. Int J Cancer.

[19] Yin T, Cho SJ, Chen X (2013). RNPC1, an RNA-binding protein and a p53 target, regulates macrophage inhibitory cytokine-1 (MIC-1) expression through mRNA stability. J Biol Chem.

[20] Chitnis MM, Yuen JS, Protheroe AS, Pollak M, Macaulay VM (2008). The type 1 insulin-like growth factor receptor pathway. Clin Cancer Res.

[21] Bach LA, Fu P, Yang Z (2013). Insulin-like growth factor-binding protein-6 and cancer. Clin Sci (Lond).

[22] Rocha RL, Hilsenbeck SG, Jackson JG, Lee AV, Figueroa JA, Yee D (1996). Correlation of insulin-like growth factor-binding protein-3 messenger RNA with protein expression in primary breast cancer tissues: detection of higher levels in tumors with poor prognostic features. J Natl Cancer Inst.

[23] Rocha RL, Hilsenbeck SG, Jackson JG, VanDenBerg CL, Weng Cn, Lee AV, Yee D (1997). Insulin-like growth factor binding protein-3 and insulin receptor substrate-1 in breast cancer: correlation with clinical parameters and disease-free survival. Clin Cancer Res.

[24] Natsuizaka M, Kinugasa H, Kagawa S, Whelan KA (2014). IGFBP3 promotes esophageal cancer growth by suppressing oxidative stress in hypoxic tumor microenvironment. Am J Cancer Res.

